# Presumptive Prostate Cancer Presenting as Low Back Pain in the Chiropractic Office: Two Cases and Literature Review

**DOI:** 10.7759/cureus.30575

**Published:** 2022-10-22

**Authors:** Eric C Chu, Robert J Trager, Colin R Lai, Benson K Leung

**Affiliations:** 1 Department of Chiropractic, New York Medical Group, Kowloon, HKG; 2 Department of Chiropractic, Connor Whole Health, University Hospitals Cleveland Medical Center, Cleveland, USA; 3 College of Chiropractic, Logan University, Chesterfield, USA

**Keywords:** male, aged, neoplasm metastasis, low back pain, prostatic neoplasms, chiropractic

## Abstract

Prostate cancer is a common type of cancer in men and may metastasize to the spine and pelvis, causing back and/or radicular pain that appears to be musculoskeletal. This presents a diagnostic challenge and can be complicated by a lack of routine screening for prostate cancer.

In two similar cases, elderly males (ages 78 and 82) with no known history of cancer and no previous prostate-specific antigen screening presented to a chiropractor with chronic, worsening radiating low back pain. In each case, a previous provider obtained radiographs and ascribed symptoms to a non-cancerous etiology (i.e., lumbar spondylosis, osteoporotic compression fracture), treated with nonsteroidal anti-inflammatory medications and physiotherapy. Given each patient’s progressive worsening and neurologic deficits, the chiropractor ordered lumbar magnetic resonance imaging, revealing potential spinal metastasis. The chiropractor referred each patient to an oncologist who performed additional testing, making a presumptive diagnosis of prostate cancer. A literature review identified seven cases of previously undiagnosed prostate cancer presenting to a chiropractor. Including the current cases, patients were often older, presenting with thoracolumbar pain caused by spine or pelvic metastasis.

The current cases and literature review illustrate that men with undiagnosed metastasis from prostate cancer may present to chiropractors complaining of spinal pain. Chiropractors should be aware of red flags warranting imaging such as older age and new or progressive symptoms and should refer patients to an oncologist when suspecting prostate cancer.

## Introduction

Prostate cancer is the second most common type of cancer in men and the fifth most common type of cancer mortality globally, with an increasing incidence in older age [1]. Prostate cancer is second only to breast cancer as a type of cancer leading to spine metastasis [2], and when this occurs, it tends to involve the thoracolumbar and sacral regions [3,4]. Accordingly, prostate cancer has been emphasized as an important differential diagnosis to consider in males over age 50 with new-onset low back pain [5].

Prostate cancer screening with prostate-specific antigen (PSA) testing is controversial, in part due to lack of evidence of significant mortality reduction [6]. As a result, guidelines regarding PSA screening vary internationally [7]. In some countries, a more comprehensive combination of PSA testing and digital rectal examination, followed by imaging-guided biopsy when indicated, is considered a gold standard for prostate cancer screening and is recommended for males aged 55 and older [7-10].

While prostate cancer has been considered less frequent in Asian countries (e.g., China, Vietnam) compared to North American countries (e.g., the United States, Canada), this may be an artifact of low rates of PSA screening in Asian countries [1,11]. A survey in 2021 revealed that in the setting of the current case, Hong Kong Special Administrative Region (HKSAR), only 5% of men with a mean age of 54 had undergone PSA screening [12]. Researchers have suggested that limited PSA screening in Asian countries could allow patients with prostate cancer to go undetected until a more advanced phase of the disease, in particular those without readily identifiable genitourinary symptoms [11].

A review of studies of patients with prostate metastasis to the spine reported that 7-19% of patients presented with no known history of cancer [4]. Patients with spinal prostate metastasis typically present with back pain (93% of cases), followed by nerve root pain (66%), and less often have neurologic deficits (20-25%) or bladder dysfunction (3%) [4]. Considering that low back pain is the most common reason that patients seek chiropractic care [13], these patients could present to a chiropractor, not realizing their symptoms are related to prostate cancer.

Evidence suggests that serious pathology such as cancer is uncommon in the chiropractic setting, affecting around 0.5% of patients with low back pain [14]. In one survey, United States chiropractors reported encountering a patient with undiagnosed cancer on average only once every eight years in practice [15]. Regardless of the rarity of these presentations, chiropractors must be prepared to recognize such patients and refer them for medical care given the potential for mortality due to undetected, untreated cancer.

Considering that chiropractors routinely evaluate patients with low back pain and rarely these patients may have undiagnosed cancer, we present two such cases, which illustrate the importance of a careful history, examination, radiological assessment, and referral for patients with prostate cancer metastasis to the spine.

## Case presentation

Case 1

A 78-year-old Asian man presented to a chiropractor in HKSAR reporting a five-year history of constant low back pain, 7/10 on the numeric rating scale, with associated episodic left hip pain. As a retired businessman, the patient had engaged in weightlifting and hiking for the past 15 years since retirement. He noted that symptoms began and progressively worsened after a hiking trip and were exacerbated by weightlifting and jogging. He denied prior trauma, weight loss, or systemic illness, yet endorsed increased urinary frequency, especially at night. The patient was a non-smoker, did not drink alcohol, and had no family history of cancer. The patient’s World Health Organization Quality-of-Life (WHOQOL) score was recorded as 60%. He had not undergone PSA screening.

**Figure 1:**
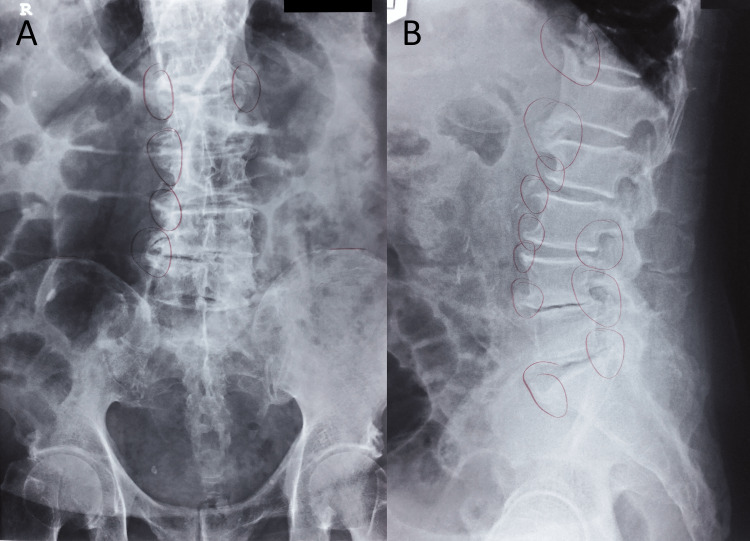
Lumbar radiographs: anteroposterior (A) and lateral (B) views. These radiographs were taken at the patient’s orthopedic re-evaluation in 2020, soon before he presented to the chiropractor, and were reported to reveal worsening of the lumbar spondylosis. The patient’s orthopedist drew markings (circles) on the film indicating areas of spondylosis.

The patient had previously seen an orthopedist who performed lumbar radiography four years prior in 2016, which revealed prominent osteophytes of the lumbar spine. The patient was treated using oral nonsteroidal anti-inflammatory medications (ibuprofen and etoricoxib) and referred to physiotherapy, which provided relief at the time. In 2020, his symptoms significantly worsened, involving radiation of pain to the sacral region. The patient returned to the orthopedist, who again ordered lumbar radiographs, revealing worsening of the lumbar spondylosis (Figure 1). His symptoms were ascribed to degenerative changes in the lumbar spine. As continued physical therapy did not relieve his symptoms, the patient sought a chiropractor for a second opinion.

On examination by the chiropractor, the patient had normal active lumbar range of motion, which did not exacerbate pain. However, passive lumbar extension and left lateral lumbar flexion exacerbated his low back and left hip pain, while passive right lateral lumbar flexion exacerbated his low back pain only. A neurological examination revealed 4/5 strength of dorsiflexion of the left ankle (Medical Research Council Scale), and otherwise preserved lower extremity muscle stretch reflexes and strength. Motion palpation revealed stiffness and tenderness at the L4/5 and L5/S1 segments. Straight leg raising and flexion, abduction, and external rotation testing of the hips did not reproduce his symptoms.

The chiropractor’s working diagnosis was lumbar stenosis; however, the differential diagnosis included malignancy given the patient’s red flags including older age and progressive worsening with neurologic deficits despite previous conservative care. Accordingly, the chiropractor ordered an MRI of the lumbar spine on the first day of consultation. In addition, the chiropractor provided a brief treatment consisting of low-force, non-thrust spinal manipulation, and therapeutic ultrasound, which were well tolerated, providing some relief.

**Figure 2:**
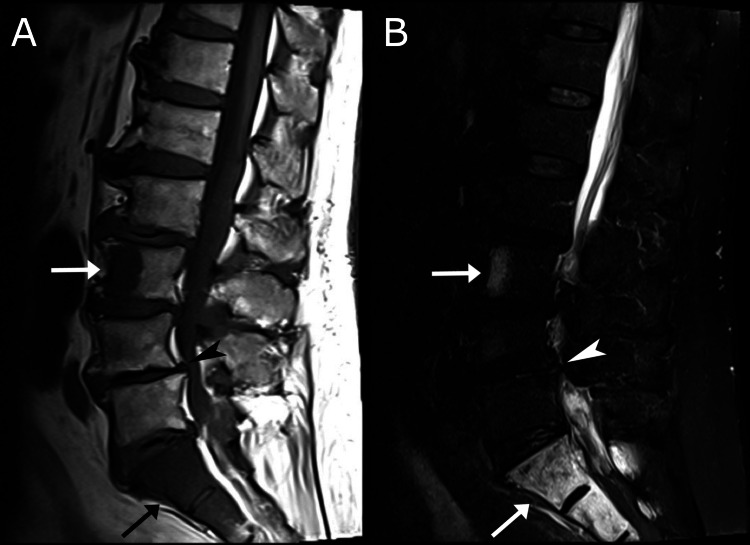
Lumbar magnetic resonance imaging (MRI), T1-weighted (A) and STIR (B) mid-sagittal view. A grade 1 L4/5 degenerative anterolisthesis (arrowhead), and disc-osteophyte complexes at L4/5 and L5/S1 are evident. Marrow replacement (arrows) is evident at the anterior region of L3 and upper sacrum, with a hypointense signal on T1 (A) and hyperintense signal on the STIR image (B), suggestive of metastasis. STIR, short tau inversion recovery

Lumbar MRI was obtained the next day, which revealed a grade I L4/5 degenerative spondylolisthesis, and disc-osteophyte complex and bilateral facet hypertrophy at L4/5 and L5/S1 causing severe spinal canal stenosis and compression of the cauda equina. In addition, marrow replacement was noted in the anterior region of L3 and sacrum (Figure 2) and was also apparent in the T10 vertebral body on the localizer view. The radiologist suggested the possibility of metastasis and multiple myeloma and recommended follow-up imaging with positron emission tomography/computed tomography (PET/CT).

The chiropractor immediately referred the patient to an oncologist. Over the subsequent month, the oncologist performed PET/CT and PSA testing, which were both abnormal, and diagnosed the patient presumptively with stage IV prostate cancer with metastasis to the spine. After discussing treatment options with the oncologist, the patient chose to forego biopsy, radiation, and chemotherapy, instead electing for watchful waiting and symptom management. He preferred to avoid an intensive treatment regimen in his older age and instead returned to the chiropractor hoping to obtain relief from his low back and hip pain.

The chiropractor treated the patient once per week for two months. Instrument-assisted soft tissue manipulation was performed by applying a lubricant to the patient’s low back and then repeatedly and gently stroking the skin surface of the overlying lumbar paraspinal muscles with a massage tool (Strig, Korea). Low-impact spinal stabilization rehabilitation exercises were also demonstrated and recommended to be performed at home daily for two to three minutes each. These included the cat-camel exercise, which is performed in the quadruped position and involves alternating positions of spinal flexion and extension, and the dying bug exercise, which is performed supine and involves raising and then lowering the opposing upper and lower extremities in an alternating fashion. Treatments provided were gentle, which was a precaution taken against potential pathological fracture given the patient’s known spinal metastasis.

At the end of the two months of care, the patient’s lumbar range of motion had returned to normal, his WHOQOL score improved to 86%, and his low back pain was intermittent and varied from 2-3/10 on the numeric pain rating scale. The patient noted that he had resumed most of his usual daily activities and exercises; however, he had stopped hiking. He enjoyed a daily morning walk in the local park, went grocery shopping regularly, and occasionally provided child care for his grandchildren. He was advised to continue performing rehabilitative exercises at home. In the sixth month after prostate cancer diagnosis, the patient succumbed to a pulmonary infection and passed away.

Case 2

An 82-year-old Asian man presented to a chiropractor in HKSAR reporting a one-year history of constant low back pain, rated 8/10 severity on the numeric pain rating scale, with associated bilateral lower extremity numbness. Symptoms had progressively worsened since falling on his buttocks four months prior. The patient reported that his symptoms were exacerbated by prolonged walking. He was currently treated for hypertension with an angiotensin-converting enzyme (ACE) inhibitor, had a remote history of urolithiasis, was a non-smoker, and had no family history of cancer. He endorsed having chronic constipation and stomach pain.

**Figure 3:**
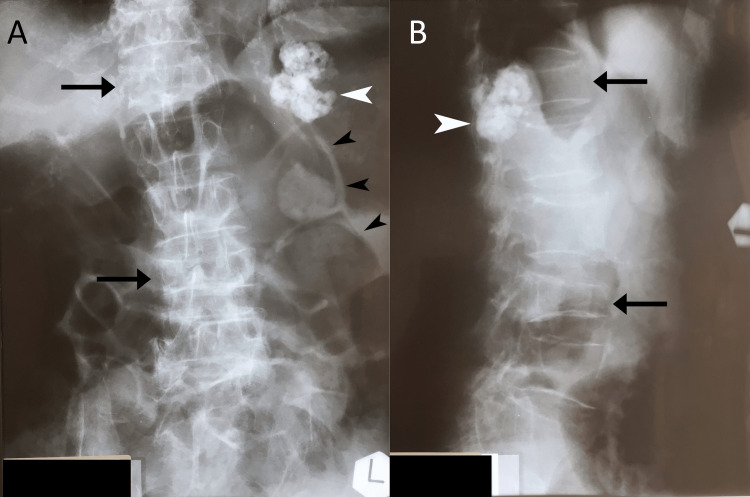
Lumbar radiographs taken prior to presentation to the chiropractor. Anteroposterior view (A) and lateral view (B). Both views illustrate a large staghorn calculus of the left kidney (white arrowhead) and compression fractures of the L3 and T12 vertebral bodies (arrows). Marked gaseous dilation of bowel loops are visible on the anteroposterior view (black arrowheads). The T12 compression fracture was not noted on the patient’s radiology report.

He had seen an orthopedist three months previously who ordered lumbar radiography, which revealed compression fracture of the L3 vertebral body, marked gaseous dilation of bowel loops, prominent osteophytes of all lumbar vertebrae, and a large staghorn calculus of the left kidney (Figure 3). The patient was considered to have an osteoporotic compression fracture and was treated using oral nonsteroidal anti-inflammatory medications (etoricoxib and celecoxib); he was also referred to physiotherapy, which provided temporary relief over a course of two weeks. The patient subsequently tried acupuncture, but his symptoms worsened and he developed weakness of the lower extremities. He then sought a chiropractor for a second opinion.

On examination by the chiropractor, the patient’s active lumbar range of motion was normal and did not exacerbate symptoms; however, passive lumbar extension elicited pain, which radiated to his left gluteal region. A neurological examination revealed 4/5 strength of the quadriceps bilaterally. Motion palpation revealed restriction and elicited tenderness at the L4/5 and L5/S1 segments. Straight leg raising, and flexion, abduction, and external rotation testing of the hips were painless.

The chiropractor’s working diagnosis was lumbar stenosis; however, malignancy was also considered. Given the patient’s red flags for serious pathology including advanced age, recent fall, compression fractures, worsening neurological signs and symptoms, gastrointestinal symptoms, and dilated loops of bowel, the chiropractor ordered MRI of the lumbar spine and abdomen at the initial consultation on an urgent basis, which was performed on the same day within the facility’s imaging center.

**Figure 4:**
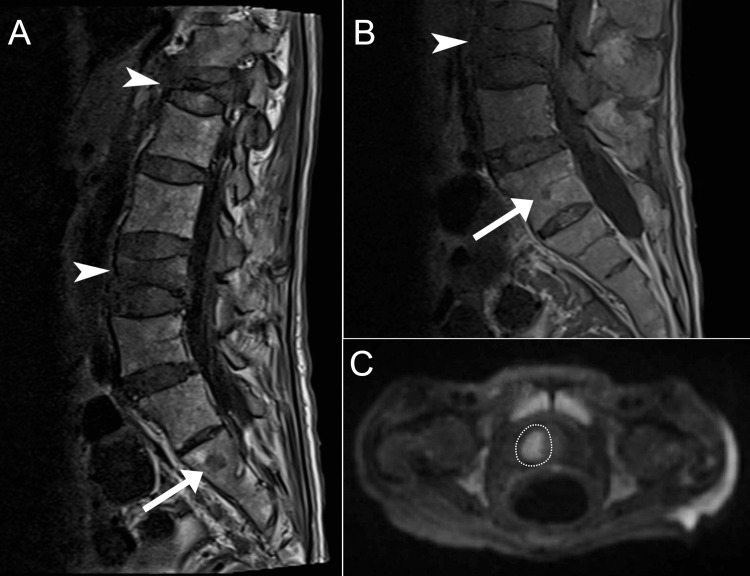
Magnetic resonance imaging of case 2. Lumbar spine MRI T1-weighted images (sagittal, A; parasagittal, B) showing compression fractures of T12 and L3 (arrowheads) and hypointense lesions in L5 and S1 (arrows), suggestive of metastasis. The diffusion-weighted axial pelvic MRI (C) shows a 2.2x1.6-cm area of marked diffusion restriction in the right basal anterior transition zone of the prostate (dotted line).

Lumbar MRI revealed T1-weighted hypointense lesions in the L1, L5, and S1 vertebral bodies of 0.7, 0.4, and 0.9 cm in diameter, respectively, collapse of the T12 vertebral body with retropulsed bone causing spinal canal stenosis, and partial collapse of the L3 vertebra (Figures 4A, 4B). The radiologist suggested that these findings were compatible with either benign vertebral compression fractures or metastasis and recommended follow-up with further imaging (i.e., PET). The patient’s abdominal MRI revealed concentric mural thickening at the distal sigmoid colon, suggestive of malignancy, as well as a lobular lesion of the spleen. The prostate was noted to be enlarged to 4.0x4.7x4.4 cm (43.3 milliliters) and contained an area of marked diffusion restriction (Figure 4C). This was suggested to represent a Prostate Imaging-Reporting and Data System (PI-RADS) 5 lesion with a very high likelihood of clinically significant cancer, and the radiologist recommended further imaging of the prostate.

The chiropractor immediately referred the patient to an oncologist who conducted PSA testing, which was reportedly elevated, which supported a presumptive diagnosis of prostate cancer. The oncologist urgently referred the patient to a surgeon who saw the patient the next day, with the aim of obtaining a colonoscopy and prostate biopsy before initiating care. However, the surgeon noted that the patient’s bowel was markedly distended and instead referred him to the emergency department that day. The patient passed away one week later at the emergency department.

Written informed consent was obtained from both patients to publish their case report and any accompanying images. In case 2, this was done prior to referral to the oncologist, and further follow-up details and consent were obtained from the patient’s next of kin.

## Discussion

The two current cases have several similarities. Each patient was an elderly male who had no prior PSA screening, had previously seen other providers, had lumbar radiography, and was treated for a non-neoplastic condition. The chiropractor recognized red flags in each patient’s history, which warranted advanced imaging, immediately ordered MRI, which was suggestive of spinal metastasis, and referred the patients to an oncologist who provided a presumptive diagnosis of prostate cancer.

The current cases highlight potential pitfalls in the diagnosis of prostate cancer and need for increased awareness of this condition among providers treating spinal disorders. In each case, a lack of baseline PSA testing conceivably delayed the diagnosis of prostate cancer. Plain radiography is not as sensitive to spinal metastasis as MRI [16]; thus, reliance on this form of imaging may have also contributed to a diagnostic delay. Furthermore, in case 1, symptoms were ascribed to degenerative spinal changes; however, these are common in the elderly population and not necessarily symptomatic [17].

Although previous research regarding isolated screening with PSA testing has yielded mixed results [6], a comprehensive clinical approach may help prevent mortality and improve quality of life in patients with prostate cancer. This includes attention to red flags [18], a thorough physical examination, and laboratory [12] and radiological investigations when indicated [4]. However, clinicians should also be mindful of their countries’ guidelines for prostate cancer screening, as recommendations regarding PSA testing, digital rectal examination, and other procedures vary internationally [7].

The primary role of the chiropractor in the current cases was to diagnose and refer each patient; however, the chiropractor appeared to provide the patient in case 1 with limited relief from his low back pain. There is a lack of guidelines and evidence regarding chiropractic care for patients with cancer [19,20]. Although high-velocity, low-amplitude spinal manipulation (i.e., involving a thrust or impulse) is contraindicated in regions of vertebral metastasis [21], certain low-force manual therapies and exercises could be safe and appropriate when carefully considered [20]. Further research is needed to examine the utility of these therapies in patients with cancer.

A literature search of PubMed, Google Scholar, and Index to Chiropractic Literature, using the search terms “chiropractic,” “chiropractor,” “prostate,” and “prostatic,” and hand-searching of review articles [19,20,22] on May 3, 2022, revealed seven previously published cases of men with previously undiagnosed prostate cancer presenting to a chiropractor, who received a presumptive or biopsy-confirmed diagnosis or prostate cancer [23-29] (Table 1). Including the current cases, this yielded a total of nine cases, with a mean age of 68±9 years. The most common presenting complaint was thoracic or lumbar pain in 7/9 (78%) cases. Diagnostic methods that initially helped uncover the cancer diagnosis varied, including digital rectal examination, laboratory testing, radiography, and MRI, as in the current cases. No patients reported a history of an elevated PSA or abnormal digital rectal examination [30].

**Table 1:** Summary of cases of undiagnosed prostate cancer presenting to chiropractors. Abbreviations: BMI, body mass index; CRP, C-reactive protein; DRE, digital rectal examination; NR, not reported; PAP, prostatic acid phosphatase; PSA, prostate-specific antigen

Author, year	Patient age	Symptoms	Previous PSA test	Risk factors or red flags	Initial test(s) suggesting diagnosis	Metastasis
Current case, 2022	78	LBP, left hip pain, polyuria, nocturia	None	Progressive worsening	Lumbar MRI	Lumbar spine
Current case, 2022	82	LBP, lower extremity numbness, constipation stomach pain	None	Progressive worsening	Lumbar and abdominal MRI	Lumbar spine, possibly other regions
Howe and Krauss, 1986 [25]	60	Left thigh pain	NR	NR	Abnormal DRE, elevated PAP and CRP, radiography	Lumbar spine, ilia
Johnson, 1994 [23]	79	LBP, polyuria, nocturia	NR	African descent, BMI 26	Abnormal DRE, elevated PSA	Thoracic spine, left pelvis
Johnson, 2010 [27]	65	LBP, constipation	Normal 2 years prior	African descent, 1 pack-year smoking, BMI 28.2	Elevated PSA	Thoracic spine
Lishchyna and Henderson, 2004 [26]	55	LBP, right hip pain	NR	Chronic, recurrent symptoms	Radiography including pelvis	Ilium
Oliver et al., 2007 [28]	58	Lower thoracic pain	NR	Lack of response to conservative care	Thoracic spine radiographs	Thoracic spine
Shorten and Kettner, 1998 [24]	66	Upper extremity weakness, paresthesia (cervical radiculopathy)	NR	Progressive worsening with neurologic deficits	Elevated alkaline and acid phosphatase, cervical spine radiographs	Cervical, thoracic, lumbar, and sacral regions
Stavish, 1990 [29]	70	LBP, urinary hesitancy, nocturia	NR	Lack of response to conservative care, unintentional weight loss	Abnormal DRE	Pelvis

The current two cases are like those previously published in that both patients were older males, were suffering from low back pain, had no previous PSA screening, and were ultimately diagnosed with prostate cancer metastasis to the spine. However, the current two cases differ in that MRI was performed earlier during evaluation. Although this difference may be because other cases were published several years ago, or described care in areas in which chiropractors may not order MRI, the current two cases highlight the utility of early MRI in identifying spinal metastasis [16]. In general, chiropractors have traditionally relied more on the radiographs than MRI when performing imaging for patients with spinal complaints [13,30]. However, MRI is the most appropriate imaging test for patients with low back pain when there is clinical suspicion of cancer [16,30,31].

Aside from these case reports and more general survey data regarding patients with cancer presenting to chiropractors [15,32], the true frequency at which patients with undiagnosed prostate cancer present to chiropractors is unclear and thus could be underreported. Given the risk for pathological fracture, cancer mortality, and other adverse outcomes when spinal metastasis is not identified, chiropractors should remain vigilant in identifying such patients.

Strengths and limitations

The current cases were strengthened by detailed clinical and imaging findings. The current report is also supported by a literature review that provides a summary of similar cases of patients with undiagnosed prostate cancer presenting to chiropractors and reveals several similarities between the current cases and others.

However, the presented case reports may not be broadly generalizable. The chiropractic scope of practice varies by country, and HKSAR chiropractors are portal of entry providers and can order MRI. Chiropractors who do not have the authority to order MRI could alternatively refer patients when imaging is indicated. Presentation of these cases to a chiropractor could be less likely in regions which prostate cancer is routinely screened for using PSA testing, although the overall value of this screening may be controversial. In HKSAR, digital rectal examination is not within the chiropractic scope of practice, which may have guided the management strategy toward advanced imaging. In both cases, unavailable baseline PSA testing and an inability to conduct digital rectal examination precluded the clinician from utilizing the Prostate Cancer Prevention Trial Prostate Cancer Risk Calculator to assess each patient’s risk of prostate cancer. In case 1, the results of follow-up PET/CT and PSA testing was unavailable. In both cases, there was limited available information regarding the exact cause of death.

## Conclusions

Men with undiagnosed prostate cancer, especially when elderly, may present to chiropractors as this type of cancer may metastasize to the thoracolumbar and pelvic regions, causing back and/or radicular pain. Chiropractors should recognize the limitations of radiography and utility of red flags warranting advanced imaging, which may facilitate a timely diagnosis in men at risk of or suspected of prostate cancer. Although the chiropractic management strategy may vary per country depending on the scope of practice including digital rectal examination, ability to order advanced imaging, or PSA screening being routine, chiropractors should refer patients to an oncologist when clinical and/or imaging findings are suggestive of cancer.
